# The relationship between health life style and spermogram Indicators among infertile men: preliminary data

**DOI:** 10.1186/s13104-020-05102-5

**Published:** 2020-06-09

**Authors:** Leila Amini, Mahsa Kahrobaie, Leila Amiri-Farahani, Hamid Haghani

**Affiliations:** 1 School of Nursing and Midwifery, Iran University of Medical Sciences, Tehran, Iran; 2 School of Health Management and Information Sciences, Iran University of Medical Sciences, Tehran, Iran

**Keywords:** Male infertility, Lifestyle, Health promotion

## Abstract

**Objective:**

Inappropriate life style has destructive effects on sperm quality and, male fertility, so that lifestyle modification may improve spermogram indexes preliminary data. This study aimed to determine the relationship between health life style and spermogram Indicators among infertile men. This analytical descriptive cross-sectional study was conducted on 199 infertile men. The data were collected through the socio-demographic and Health Promoting Lifestyle Profile questionnaires Descriptive statistics independent t-test and Pearson correlation were used to analyze the data through SPSS.

**Results:**

The mean (standard deviation) of total score of the health promoting lifestyle was (2.39 ± 0.39). The highest mean score was in Health Responsibility subscale (2.51 ± 0.52) and the lowest mean score was in the nutrition subscale (2.24 ± 0.44). Stress management showed significantly correlated with sperm morphology (p = 0.025). Also, spiritual growth with the Sperm concentration (p < 0.001), and sperm motility (p = 0.004) were statistically correlated, and health responsibility dimensions were statistically correlated with the Sperm concentration (p = 0.003) and sperm motility (p = 0.002). Considering that the mean of total score of the health promoting lifestyle and its correlation with some of spermogram indicators shows a need for improving lifestyle in infertile men who referred to infertility clinics.

## Introduction

Although infertility is not a disease; it can cause disturbances in people’s lives because it affects every aspect of a person’s life [1]. According to world statistics, the prevalence of infertility among couples is 15% [2]. The prevalence of infertility in different parts of Iran has been reported differently, but the overall average of infertility in Iran is 13.2% [3]. The rate of male factor infertility has been growing faster than that of female factor infertility in recent decades [4]. Semen quality is one of the most important determinants of infertility in men. The prevalence of infertility in men has been increasing due to the decline in semen quality in recent decades [5]. The results of recent studies confirm a decrease in semen quality which has led to an increased willingness to conduct research on the effect of lifestyle on male fertility [6]. Inappropriate lifestyles have detrimental effects on sperm quality [7] and consequently male fertility [8] so that lifestyle change can improve spermogram parameters [9]. Improving lifestyle components can help people cope with daily stresses, and having a healthy lifestyle can play a role in happiness and preventing stress. Psychological stress itself has been recognized as one of the contributing factors in idiopathic male infertility [10]. In general, considering that one of the essential strategies for promoting health is to adopt a healthy lifestyle, as well as the importance of this subject and the limited information on the life style of Iranian infertile men, and given that all research in the area of relationship between spiritual growth and infertility has been conducted in the women’s domain and studies on the relationship between spirituality, religion and fertility have not been conducted in men; therefore, the present study was conducted to determine the relationship between health lifestyle and spermogram indexes in infertile men referred to the urology clinic in Tehran.

## Main text

### Methods

The present study is a cross-sectional study. The study population included all infertile men referred to the comprehensive urology clinic in Tehran in 2018. The study sample was considered as 199 individuals at 95% confidence level and 80% test power, assuming that the correlation coefficient between health lifestyle with each of the spermogram indices in infertile men to be 0.2 and this correlation to be considered statistically significant.

Sampling was conducted on a continuous basis. The study inclusion criteria included men with Iranian nationality, 20–50 years old, not working in high-risk jobs in terms of effects on spermograms (such as building painters, personnel of radiology, radiotherapy, nuclear energy, etc.), not using infertility-related medication during the past 3 months, no Incidence of psychological illness according to the patient’s statement or record of the patient, not having urinary tract infection or genital infection, ability to read and write, and no history of varicocele surgery.

Data collection tools included demographic questionnaires, spermogram indices data sheet and health promoting lifestyle questionnaire which were completed by the participants in the study.

The Health Promoting Lifestyle Questionnaire [11], which has an English version of 52 items, is responded with a 4-point response format (1 = Never, 2 = Sometimes, 3 = often, and 4 = routinely). The tool measures health-promoting behaviors in six dimensions: nutrition, Physical Activity, health responsibility, stress management, Interpersonal Relations, spiritual growth.

Scoring for the Healthy Lifestyle was calculated using an average of 52 questions for each of the 6 scales. The tool has been translated into several languages and its validity and reliability have been confirmed.

Two-week test–re test method was used for reliability of the instrument which was 0.91 for overall profile and ranged from 0.71 (spiritual growth) to 0.89 (nutrition). The present study was approved by the Research Council of Iran University of Medical Sciences after receiving the Code of Ethics (IR.IUMS.REC 1396,9413373005) from the Ethics Committee of Iran University of Medical Sciences. Complete descriptions of the purpose and procedure of the study were also provided to the individuals during the sampling and they were assured of the confidentiality of all information. Finally, written informed consent was obtained from the samples.

Statistical analysis was performed using SPSS 20. Descriptive statistics including frequency distribution as well as central and dispersion indices such as mean and standard deviation were used to describe health promoting lifestyle and demographic characteristics. T-test and Pearson’s correlation coefficient were used to determine the relationship between lifestyle characteristics and spermogram indices. Significance level for statistical tests was considered less than 0.05.

### Results

The highest percentage of participants was in the age group of 31–40 years (64.8%) and the lowest percentage was in the age group of 41–50 years (16.3%). The mean age of the participants in this study was 35.46 ± 5.69 with a maximum-minimum of 20–50 years. Other demographic characteristics of the participants are shown in Table 1.

**Table 1:** Demographic, social and fertility characteristics of infertile men and their spouses referred to the comprehensive urology clinic in Tehran

Characteristic	Frequency (percentage)		Characteristic	Frequency (percentage)	
BMI	18/5–24/9	45 (23)	Age (year)	20–30	37 (18·9)
				40–31	127 (64·8)
				41–50	32 (16·3)
	25–29/9	95 (48·5)			
	30–34/9	44 (22·4)	Job type	Employe	84 (44)
	35–39/9	10 (5·1)		Freelance job	62 (32·5)
	40 >	2 (1)		Others	45 (23·5)
Level of education	Diploma and lower diploma level	82 (41·8)			
	Associate Degree and Masters	71 (36·2)			
	Master’s Degree and higher	43 (21·9)			

The mean and standard deviation of the overall health promoting lifestyle score of the samples were equal to 2.39 ± 0.39 with a domain of 1.52–3.58 from the obtainable limit of 1–4. Highest score on health responsibility dimension (2.51 ± 0.52) with maximum domain of 1.22 ± 3.78 and lowest score on nutrition dimension (2.24 0 0.44) with domain of 1.22 ± 3.44 was observed (Table 2).

**Table 2:** Numerical indices of dimensions and total score of health promoting lifestyle in the studied units

Dimensions and total score of health lifestyle	SD	M	Minimum	Maximum
Nutrition	0·44	2·24	1·22	3·44
Physical activity	0·52	2·42	1·38	3·75
Stress management	0·49	2·47	1·13	3·70
Health responsibility	0·52	2·51	1·22	3·78
Spiritual growth	0· 51	2·35	1	3·98
Interpersonal relations	0·43	2·25	1·22	3·44
Total score of health lifestyle	0·39	2·39	1·52	3·58

Based on Pearson correlation and t-test, there was a significant statistical relationship between the dimensions of responsibility for health and spiritual growth with all three spermogram indices (p < 0.05). In stress management dimension, significant relationship was observed only with sperm morphology (p-value = 0.003) (Table 3). No significant relationship was found between other dimensions of life style and spermogram indices.

**Table 3:** The relationship between health life style and spermogram Indicators among infertile men

Lifestyle and its dimensions spermogram indicators	Lifestyle	Nutrition	Stress management	Physical activity	Interpersonal relationships	Spiritual growth	Health responsibility
Sperm count	r = 0·014 p-value = 0·046	r = 0·065 p-value = 0·35	r = 0·12 p-value = 0·074	r = 0·037 p-value = 0·611	r = 0·01 p-value = 0·88	r = 0·41 p-value < 0·001	r = 0·20 p-value = 0·003
Sperm morphology	r = 0·06 p-value = 0·036	r = 0·75 p-value = 0·29	r = 0·16 p-value = 0·025	r = 0·017 p-value = 0·81	r = 0·05 p-value = 0·48	r = 0·11 p-value = 0·12	r = 0·08 p-value = 0·22
Sperm motility	r = 0·012 p-value = 0·08	r = 0·53 p-value = 0·45	r = 0·10 p-value = 0·13	r = 0·09 p-value = 0·21	r = 0·073 p-value = 0·30	r = 0·20 p-value = 0·004	r = 0·22 p-value = 0·002

## Discussion

This study seemed to be the first study on infertile men in Iran designed to determine the relationship between infertile men’s lifestyle and sperm parameters. Results of this study showed that the overall score of health promoting lifestyle was 2.39 ± 0.39 with minimum–maximum of 1.52–3.58. The highest score in health responsibility dimension (2.51 ± 0.52) with minimum–maximum of 1.22–3.78 and lowest score in nutrition dimension (2.24 ± 0.44) with minimum–maximum of 1.22–3.44 was observed. A similar study conducted by Mirghafourvand et al. [12] on the relationship between health promoting behaviors and demographic characteristics in infertile couples in 2013. The results showed that among the health promoting behaviors in men, the lowest score was related to physical activity and health responsibility, both of which had the mean value of 2.3 and standard deviation of 0.5 and the highest scores were related to nutrition and spiritual growth with the mean value of 2.6 and standard deviation of 0.5 in both. In this study, education level and income were predictive of health promoting behaviors. However, this study only examined the relationship between sperm parameters and lifestyle. In this study, there was no relationship between spermogram indices and the first dimension (nutrition) with the mean of 2.24, the second dimension (physical activity) with the mean of 2.42 and the fifth dimension (interpersonal relationships) with the mean of 2.25. The low average scores on these dimensions indicate the need for training and awareness of men in these areas. Regarding the third dimension (stress management), it was observed that stress management with mean of 2.47 in men with normal sperm morphology was significantly higher than that of men with abnormal sperm morphology. Since there was no study on the relationship between stress management and spermogram indices, the effects of stress on spermogram indices were investigated.

A study by Janevic et al. on stress and sperm quality showed that men who experienced two or more stressful events in their lives over the past year had a lower percentage of normal sperm motility and morphology compared to men who were not exposed to stressful events. But there was no difference in the number of sperms between the two groups [13]. The results of the present study were not significantly different from those of Janevic et al. Although stress management in males is not in a desirable level in this study; there is a direct relationship between mean stress management score and sperm morphology indicating the desirable effects of stress management on sperm indices improvement.

The results related to the dimension of responsibility for health indicated that this dimension has the highest dimension with mean of 2.51 ± 0.52 and minimum–maximum of 1.22–3.78. There was also a statistically significant relationship between this dimension of lifestyle and spermogram indices. It shows that men are prepared to receive health training to achieve well-being while men’s health training can be a strong point in training other health-promoting behaviors. No study was found to compare the results of the present study with that of the relationship between health responsibility and spermogram indices, which may indicate the innovation of this study.

Concerning the answer to the last specific goal of this study, namely determining the relationship between spiritual growth dimension with quantitative and qualitative indices of sperm in infertile men, the results of t-test indicated that there was a significant relationship between spiritual life style and all three spermogram indices (p-value < 0.05). A review study by Zimmer et al. [14] suggested that religion can reduce stressful life events by enhancing feelings of life satisfaction, optimism, confidence and a sense of social support. On the other hand, emotional stress affects the fertility of men by affecting the number and motility of sperm [10]. Findings of religion and stress management in this study provide evidence by which it can be concluded that religion can influence sperm indices through its effect on reducing anxiety. Based on what mentioned above, it seems that religion plays a less prominent role in controlling social stress because of the increasing stressors in the current society.

## Conclusion

Given the health-promoting lifestyle score and all its dimensions in the middle of the domain of obtainable scores, there seems to be a need for interventions to improve the lifestyle of infertile men referred to infertility centers to improve their lifestyles and domains.

Considering that this study was conducted for the first time in Iran and that the lifestyles of different cities are different, it is therefore necessary to conduct a multi-center study on the population of men from different regions and cultures.

## Limitations

Some limitations of the current study should be considered. Briefly, because of the cross-sectional design of this study, causal inferences could not be extracted; we were not able to declare whether spiritual growth was the reason for stress management. Therefore, our findings need to be con-firmed in future whit the bigger number of patients and than compare data and results to the group of healthy controls [11].

## Data Availability

Data can be reached by contacting the corresponding author.
